# NAFLD or MAFLD: Which Has Closer Association With All-Cause and Cause-Specific Mortality?—Results From NHANES III

**DOI:** 10.3389/fmed.2021.693507

**Published:** 2021-07-01

**Authors:** Qi Huang, Xiantong Zou, Xin Wen, Xianghai Zhou, Linong Ji

**Affiliations:** Department of Endocrinology and Metabolism, Peking University People's Hospital, Beijing, China

**Keywords:** NAFLD, MAFLD, mortality, NHANES, diagnosis

## Abstract

**Background:** The recent change of terminology from non-alcoholic fatty liver disease (NAFLD) to metabolic dysfunction-associated fatty liver disease (MAFLD) has raised heated discussion. We aim to investigate the association of MAFLD or NAFLD with all-cause and cause-specific mortality to compare the outcomes of the two diagnostic criteria in population-based study.

**Methods:** We recruited 12,480 participants from the Third National Health and Nutrition Examination Survey (NHANES III) with matched mortality data in 2015. Participants were divided into four groups for survival analysis: without NAFLD or MAFLD, with only NAFLD, only MAFLD. Cox proportional hazard regression was used to estimate multivariable-adjusted hazard ratios (HRs) and 95% confidence intervals (CIs) for all-cause and cause-specific mortality. Subgroup analysis were applied in MAFLD patients.

**Results:** The weighted prevalence of MAFLD and NAFLD was relatively 27.4 and 27.9%. Participants with NAFLD or MAFLD were largely overlapped (weighted Cohen's kappa coefficient 0.76). MAFLD increased the overall risk for total mortality in a greater magnitude than NAFLD [HR 2.07 (95% CI 1.86, 2.29) vs. 1.47 (1.20, 1.79)], However, the difference was non-significant after metabolic parameters were adjusted. Risks for cardiovascular, neoplasm, and diabetes-related mortality were similar between MAFLD and NAFLD. Referring to individuals without both NAFLD and MAFLD, individuals with only NAFLD showed reduced total mortality [HR 0.48 (0.34, 0.68)] and neoplasm mortality [HR 0.46 (0.24, 0.89)] in crude. Nevertheless, individuals with only MAFLD independently increased the risk for total mortality [adjusted HR 1.47 (1.22, 1.77)] and neoplasm mortality [aHR 1.58 (1.09, 2.28)]. The risk for overall mortality in MAFLD was consistent between subgroups except for race-ethnicity and whether secondary to viral hepatitis.

**Conclusions:** Participants with MAFLD or NAFLD were highly concordant. MAFLD showed greater risk for all-cause mortality and equal risk for cause-specific mortality referring to NAFLD. The new terminology excluded participants with lower mortality risk and included participants with higher risk. Drug development for MAFLD should consider ethnic differences.

## Introduction

Non-alcoholic fatty liver disease (NAFLD) is the most common liver disease affecting around one-quarter of the population worldwide, causing a global economic burden (1). The definition of NAFLD requires presence of fat on imaging to liver biopsy and exclusion of other liver diseases e.g., excess alcohol intake, drug-induced liver injury, and viral hepatitis (2). NAFLD is also regarded as a “metabolic disease” since it is closely associated with metabolic disorders including obesity, dyslipidemia, and diabetes mellitus (3), of which the common etiology is insulin resistance (4). Patients with NAFLD have a higher risk of cardiovascular events. The leading cause of mortality in NAFLD patients is cardiovascular disease and major excess mortality may result from extrahepatic cancer (1, 5, 6). A meta-analysis suggested that NAFLD was independently associated with increased absolute risk of all-cause mortality, but the risk for cardiovascular and cancer mortality was similar between NAFLD and non-NAFLD participants (1, 6–8).

The progression and prognosis of NAFLD are highly heterogeneous. Only 2–3% of participants progressed from steatosis to non-alcoholic steatohepatitis (NASH) and advanced fibrosis. Liver related mortality only explained 7% of deaths among NAFLD patients (9, 10). At the beginning of 2020, experts from the European Liver Patient's Association (ELPA) proposed a change of nomenclature from NAFLD to metabolic dysfunction-associated fatty liver disease (MAFLD), which was mainly defined as liver fat deposition along with obesity, diabetes, or combined metabolic disorders (11, 12). This change emphasized the importance of metabolic disorder complicated with fatty liver regardless of the heterogeneous etiology since the exclusion of other liver diseases was no longer required.

Intense dispute raised over the change of the terminology since whether the change from NAFLD to MAFLD can benefit clinical practice and drug development is largely unknown. Studies suggested that participants with NAFLD and MAFLD were highly compatible with each other, and patients with MAFLD were more likely to have worse metabolic profiles than NAFLD (13, 14). Other experts concerned that the change may exclude patients with worse outcome, such as participants with “lean NAFLD” who have lower BMI and better metabolic profile, and participants with severe hepatic steatosis who may have more liver fibrosis and elevated long-term comorbidities (13, 15). In addition, although MAFLD may reflect relevant risk factors as a metabolic disease, whether this change is necessary regarding biomarker identification, treatment strategy and prognosis is largely unknown (16).

A key question to be answered is whether the change from NAFLD to MAFLD could affect the association between fatty liver and clinical outcomes. A study from Japan suggested that individuals with NAFLD and MAFLD had similar metabolic traits at baseline as well as incidence for cardiovascular events after a 7-year follow-up (17). However, the association between MAFLD and mortality in the long run was largely unknown. Here we aimed to use the National Health and Nutrition Examination Survey III cohort and the follow-up mortality data to answer whether the terminology MAFLD is superior to NAFLD regarding their long-term mortality risk and cause-specific mortality risk.

## Materials and Methods

### Study Design and Participants

The Third National Health and Nutrition Examination Survey (NHANES III) profiles health estimates of civilian non-institutionalized US population using a multistage, stratified sampling design from 1988 to 1994 (18). Ultrasound grading of hepatic steatosis was combined at baseline. Linked mortality information through December 31, 2015, was provided by the National Center for Health Statistics (NCHS) of the Centers for Disease Control and Prevention (CDC).

**Figure 1:**
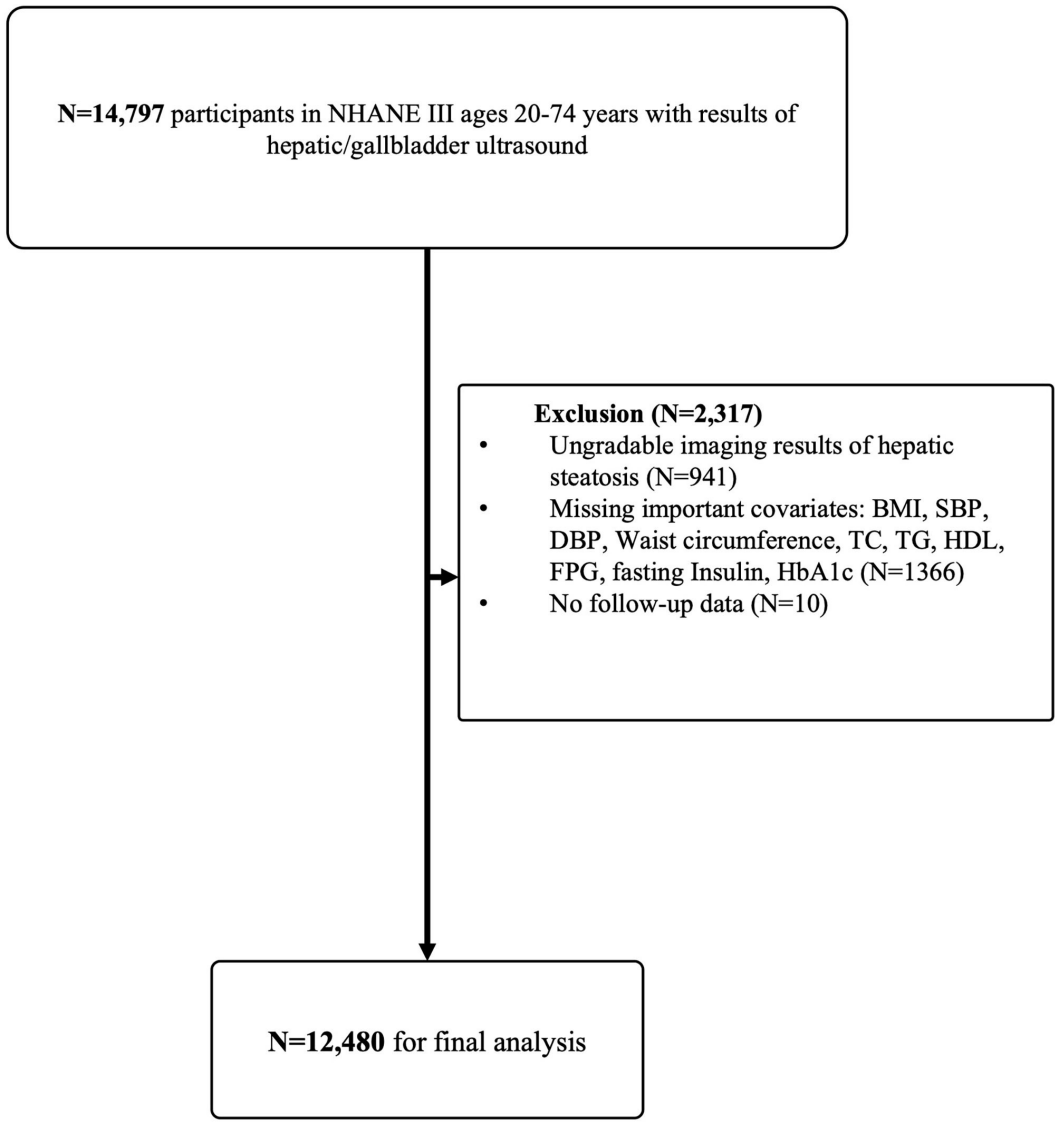
Flow-chart of the Study. NHANE III, The National Health and Nutrition Examination Survey III (1988–1994). BMI, body mass index; SBP, systolic blood pressure; DBP, diastolic blood pressure; TC, total cholesterol; TG, triglyceride; HDL, high-density lipoprotein cholesterol; FPG, fasting plasma glucose; HbA1c, glycosylated hemoglobin.

In NHANES III, 14,797 participants aged 20–74 years with assessment of hepatic steatosis were recruited. Exclusion criteria included: (1) ungradable images of hepatic steatosis (*N* = 941); (2) participants without important covariates: body mass index (BMI), systolic blood pressure (SBP), diastolic blood pressure (DBP), waist circumference, total cholesterol (TC), total triglyceride (TG), high-density lipoprotein cholesterol (HDL), fasting plasma glucose (FPG), fasting insulin and glycosylated hemoglobin (HbA1c) (*N* = 1,366); (3) participants with missing follow-up data (*N* = 10). After exclusion, 12,480 eligible participants were followed up for a medium of 22.8 years (interquartile range 20.7–24.7 years, Figure 1).

### Laboratory Measurement and Index Calculation

Serum biochemistries were measured by the Hitachi 737 automated multichannel chemistry analyzer (Boehringer Mannheim Diagnostics, Inc., Indianapolis, Indiana). Homeostatic Model Assessment for Insulin Resistance (HOMA-IR) was adopted to estimate the level of β-cell function. Methods for non-invasive fibrosis assessment, such as NAFLD fibrosis score (NFS score), AST-to-platelet ratio index (APRI), and fibrosis-4 index (FIB-4), were evaluated by original formulas (Supplementary Table 1).

### Definitions and Subgroups

Categorized assessment of hepatic steatosis by ultrasound encompassed none, mild, moderate and severe, and only mild to severe hepatic steatosis was regarded as evidence of hepatic steatosis (19). NAFLD was diagnosed if an adult with steatosis confirmed by ultrasound without (1) high alcoholic consumption (over one drink daily among women or over two drinks daily among men); (2) presence of hepatitis B surface antigens or antibodies to hepatitis C; (3) iron overload (transferrin saturation ≥ 50% along with serum ferritin ≥ 400 μg/L in women and ≥500 μg/L in men) (20). MAFLD was defined by the international expert consensus statement in 2020 (12), including ultrasound confirmed hepatic steatosis plus one of the three criteria: overweight or obesity defined as BMI ≥ 25 kg/m^2^, presence of type 2 diabetes mellitus, and metabolic disorders described by any two indicators: (1) waist circumference (WC) ≥ 102 cm in men or ≥88 cm in women; (2) blood pressure ≥140/90 mmHg or taking anti-hypertension drugs; (3) raised triglycerides (plasma triglycerides ≥ 1.70 mmol/L or taking specific anti-lipid agents); (4) reduced HDL cholesterol (plasma HDL <1.0 mmol/L for men and <1.3 mmol/L for women or taking specific agents); (5) prediabetes status (FPG 5.6–6.9 mmol/L, or 2-h post-load glucose levels 7.8–11.0 mmol or HbA1c 5.7–6.4%); (6) HOMA-IR ≥ 2.5; (7) plasma high-sensitivity C-reactive protein (CRP) level > 2 mg/L.

The presence of diabetes mellitus was defined as a self-report history of diabetes mellitus, fasting glucose levels (FPG) ≥ 7.0 mmol/L, 2-h post-load glucose levels ≥ 11.0 mmol (for participants given an oral glucose tolerance test), HbA1c ≥ 6.5% or taking diabetes drugs. Hypertension was defined as BP ≥ 140/90 mmHg, or taking anti-hypertension drugs. The definition of metabolic syndrome was according to the joint interim statement in 2009 (21). Waist circumference criteria in ATP III (≥102 cm in male; ≥88 cm in female) was used for abdominal obesity in the United States. Elevated liver enzymes were defined as AST > 37 U/L in men and >31 U/L in women or ALT > 40 U/L in men and >31 U/L in women.

According to the NCHS, all-cause death recorded all deceased participants. Main causes of death following the guidelines of International Statistical Classification of Diseases, Injuries, and Causes of Death (ICD-9 before 1998 and ICD-10 afterwards) presented as cause-specific mortality (22), consisting of cardiovascular mortality recorded by heart and cerebrovascular diseases, neoplasm mortality recorded by malignant neoplasms in all systems, and diabetes-related mortality recorded by diabetes mellitus.

We further separated the cohort into the four mutually exclusive groups based on definitions of MAFLD and NAFLD. Group M+N: participants meet the diagnostic criteria for both MAFLD and NAFLD definitions were in group M+N; Group N: participants can be defined as NAFLD but not MAFLD; Group M: participants defined as MAFLD but not NAFLD in group N or M; participants excluded by both definitions were viewed as control group.

### Statistical Analysis

All analysis was weighted by sample weights to reflect population-based estimates. Continuous data were presented as mean and 95% confidence intervals (geometric mean for variables without normal distribution). Categorical variables were displayed as percentages. The baseline characteristics of the participants among groups were compared by one-way ANOVA test when appropriate for continuous variables or chisq test for categorical variables.

For survival analysis, we used Kaplan-Meier methods to estimate cumulative hazard. To establish cox regression models, the following confounders were considered initially:

- Sociodemographic features: age, sex, race-ethnicity, smoking status.
- Hepatic assessment: alanine transaminase (ALT), aspartate transaminase (AST), CRP, alkaline phosphatase (ALP), FIB-4 score, NFS score, APRI.
- Metabolic assessment: BMI, WC, SBP, DBP, FPG, fasting insulin, HOMAIR, TC, TG.

LASSO regression with minimum mean 10-fold cross-validated error was applied for confounder selection to avoid multicollinearity. Among confounders above, we excluded the variables that were penalized to zero by LASSO model (Supplementary Table 2). The LASSO model suggested waist circumference and HbA1c, were stronger indicators than BMI and diabetes, so we used the former instead. Variables with no-zero parameter were classified as above and adjusted stepwise in cox regression models to estimate hazard ratios (HR) and 95% confidence intervals (95% CI) for overall and cause-specific mortality. Participants missing relevant covariates were excluded.

Finally, we assessed the association of MAFLD with all-cause, cardiovascular, and neoplasm mortality within subgroups by age (20–39 years, 40–55 years, >55 years), sex, race-ethnicity, diabetes, hypertension, dyslipidemia (raised triglycerides or reduced HDL cholesterol), metabolic syndrome, BMI (Underweight/normal weight: <25 kg/m^2^, overweight: 25–30 kg/m^2^ and obesity: >30 kg/m^2^), severity of hepatic steatosis, NFS, APRI and FIB-4 score (≤ weighted mean value, >weighted mean value), presence of other etiologies (alcohol, hepatitis virus, and iron overload), adjusting by age, sex, and race-ethnicity if appropriate. Bonferroni correction was applied and significance was defined as *p* < 0.0033.

All statistical analyses were conducted in R software version 4.0.2. The LASSO regression model was conducted by the R-package “glmnet” (23).

## Results

### Baseline Characteristics of the Participants With MAFLD and NAFLD

Of 12,480 participants, 3,909 were diagnosed with MAFLD (weighted prevalence 27.4%) and 3,779 were diagnosed with NAFLD (weighted prevalence 27.9%) (Table 1). Correlation analysis suggested MAFLD was highly concordant with NAFLD (weighted Cohen's kappa coefficient 0.76).

**Table 1:** The characteristics of the participants (*N* = 12,480)^*^.

	**Overall**	**MAFLD vs. control**		**NAFLD vs. control**		**Separate groups**			
		**Non-MAFLD**	**MAFLD**	**Non-NAFLD**	**NAFLD**	**Control**	**N**	**M**	**M+N**
*N* (%)	12,480 (100)	8,571 (72.6)	3,909 (27.4)	8,701 (72.1)	3,779 (27.9)	8,043 (67.5)	528 (5.1)	658 (4.6)	3,251 (22.8)
Age (years)	42.1 (41.8, 42.4)	40.1 (39.8, 40.4)	47.4 (46.9, 47.9)	40.9 (40.6, 41.3)	45.1 (44.6, 45.6)	40.5 (40.2, 40.9)	34.2 (33.2, 35.1)	46.9 (45.8, 47.9)	47.5 (47.0, 48.0)
Men (%)	5,865 (48.7)	3,897 (46.2)	1,968 (55.1)	4,164 (48.4)	1,701 (49.3)	3,702 (47.1)	195 (33.7)	462 (66.7)	1,506 (52.8)
**Race-ethnicity (%)**									
Non-Hispanic White	4,648 (76.0)	3,276 (76.7)	1,372 (74.2)	3,284 (76.4)	1,364 (75.1)	3,070 (76.6)	206 (78.5)	214 (73.5)	1,158 (74.4)
Non-Hispanic Black	3,544 (10.5)	2,658 (11.0)	886 (9.2)	2,668 (11.1)	876 (9.1)	2,495 (11.1)	163 (9.8)	173 (10.5)	713 (8.9)
Mexican-American	3,765 (5.5)	2,272 (4.8)	1,493 (7.4)	2,371 (4.9)	1,394 (7.0)	2,127 (4.8)	145 (4.8)	244 (7.3)	1,249 (7.5)
Others	523 (8.0)	365 (7.5)	158 (9.1)	372 (7.6)	151 (8.9)	351 (7.5)	14 (6.9)	27 (8.7)	131 (9.3)
Ever smoking (%)	6,408 (55.3)	4,326 (54.1)	2,082 (58.3)	4,554 (55.8)	1,854 (53.8)	4,101 (54.8)	225 (44.4)	453 (70.5)	1,629 (55.9)
BMI, kg/m^2^	26.5 (26.4, 26.6)	25.0 (25.0, 25.1)	30.5 (30.3, 30.7)	25.6 (25.5, 25.7)	28.9 (28.7, 29.1)	25.3 (25.2, 25.4)	21.4 (21.2, 21.6)	29.9 (29.5, 30.4)	30.6 (30.4, 30.8)
Waist circumference (M), cm	95.0 (94.7, 95.4)	91.1 (90.7, 91.4)	104 (103, 104)	92.6 (92.2, 92.9)	101 (101, 102)	91.6 (91.3, 92.0)	81.0 (79.9, 82.2)	102 (101, 104)	104 (104, 105)
Waist circumference (F), cm	91.7 (91.4, 92.0)	84.5 (84.1, 84.8)	101 (101, 102)	86.2 (85.8, 86.6)	94.7 (93.9, 95.5)	85.5 (85.1, 85.8)	73.9 (73.3, 74.6)	102 (100, 105)	101 (101, 102)
SBP, mmHg	121 (120, 121)	118 (118, 118)	127 (127, 128)	119 (119, 120)	124 (123, 124)	119 (118, 119)	109 (108, 110)	129 (128, 130)	127 (127, 128)
DBP, mmHg	74.3 (74.1, 74.4)	72.9 (72.7, 73.1)	77.9 (77.6, 78.3)	73.6 (73.4, 73.8)	76.1 (75.7, 76.4)	73.2 (73.0, 73.4)	68.9 (68.2, 69.6)	79.2 (78.4, 80.1)	77.7 (77.3, 78)
HbA1C, %	5.32 (5.31, 5.34)	5.20 (5.19, 5.22)	5.64 (5.60, 5.67)	5.24 (5.22, 5.25)	5.54 (5.51, 5.58)	5.22 (5.20, 5.23)	5.02 (4.99, 5.05)	5.52 (5.43, 5.62)	5.66 (5.62, 5.70)
HOMA-IR	2.04 (2.02, 2.07)	1.70 (1.68, 1.72)	3.32 (3.25, 3.4)	1.81 (1.78, 1.83)	2.81 (2.74, 2.88)	1.73 (1.71, 1.75)	1.32 (1.28, 1.37)	3.35 (3.15, 3.55)	3.32 (3.24, 3.41)
TG, mmol/L	1.31 (1.30, 1.32)	1.14 (1.13, 1.16)	1.87 (1.84, 1.91)	1.2 (1.19, 1.21)	1.64 (1.61, 1.67)	1.17 (1.15, 1.18)	0.87 (0.85, 0.90)	1.79 (1.71, 1.87)	1.89 (1.85, 1.93)
TC, mmol/L	5.13 (5.11, 5.15)	5.03 (5.01, 5.05)	5.41 (5.38, 5.45)	5.09 (5.07, 5.11)	5.24 (5.21, 5.28)	5.07 (5.05, 5.1)	4.52 (4.45, 4.59)	5.37 (5.27, 5.46)	5.42 (5.38, 5.45)
HDL(M), mmol/L	1.13 (1.13, 1.14)	1.19 (1.18, 1.20)	1.02 (1.01, 1.04)	1.18 (1.17, 1.19)	1.03 (1.01, 1.04)	1.18 (1.17, 1.19)	1.29 (1.24, 1.33)	1.14 (1.11, 1.18)	1.00 (0.98, 1.01)
HDL(F), mmol/L	1.38 (1.37, 1.38)	1.43 (1.42, 1.44)	1.22 (1.21, 1.24)	1.42 (1.41, 1.43)	1.27 (1.25, 1.28)	1.42 (1.41, 1.43)	1.49 (1.46, 1.53)	1.35 (1.29, 1.4)	1.20 (1.19, 1.22)
AST, U/L^#^	21.4 (21.2, 21.6)	20.2 (20.0, 20.4)	24.5 (24.0, 25.1)	21.1 (20.8, 21.4)	22.2 (21.9, 22.6)	20.3 (20.1, 20.5)	19.9 (18.8, 20.9)	33.3 (31.1, 35.4)	22.8 (22.4, 23.2)
ALT, U/L^#^	18.0 (17.8, 18.3)	15.9 (15.7, 16.1)	23.6 (23.0, 24.2)	16.9 (16.7, 17.2)	20.8 (20.3, 21.3)	16.0 (15.7, 16.2)	14.9 (13.9, 15.8)	31.0 (28.9, 33.0)	22.1 (21.6, 22.7)
GGT, U/L^#^	29.4 (28.6, 30.1)	24.7 (24.1, 25.4)	41.6 (39.8, 43.4)	28.0 (27.1, 28.9)	32.8 (31.6, 34.0)	25.2 (24.5, 25.9)	18.4 (16.1, 20.8)	69.1 (61.1, 77.1)	36.2 (34.8, 37.6)
ALP, U/L^#^	80.9 (80.4, 81.4)	78.3 (77.7, 78.9)	87.6 (86.7, 88.6)	79.3 (78.7, 79.9)	84.8 (83.9, 85.7)	78.7 (78.1, 79.3)	72.9 (70.7, 75.2)	88.7 (85.9, 91.4)	87.4 (86.5, 88.4)
CRP, mg/L	3.93 (3.83, 4.03)	3.59 (3.47, 3.70)	4.83 (4.64, 5.02)	3.75 (3.63, 3.87)	4.38 (4.21, 4.56)	3.69 (3.56, 3.81)	2.29 (2.18, 2.41)	4.73 (4.22, 5.25)	4.85 (4.65, 5.06)
NFS score^#^	−2.23 (−2.26, −2.21)	−2.44 (−2.47, −2.41)	−1.68 (−1.73, −1.63)	−2.34 (−2.37, −2.30)	−1.97 (−2.02, −1.92)	−2.40 (−2.43, −2.36)	−3.03 (−3.12, −2.93)	−1.45 (−1.58, −1.32)	−1.73 (−1.78, −1.68)
APRI score^#^	0.22 (0.22, 0.22)	0.20 (0.20, 0.21)	0.26 (0.25, 0.28)	0.22 (0.21, 0.23)	0.22 (0.21, 0.23)	0.20 (0.20, 0.21)	0.21 (0.19, 0.23)	0.47 (0.39, 0.54)	0.22 (0.22, 0.23)
FIB-4 score^#^	0.91 (0.89, 0.92)	0.86 (0.85, 0.87)	1.03 (0.99, 1.06)	0.90 (0.89, 0.92)	0.91 (0.89, 0.93)	0.87 (0.85, 0.88)	0.77 (0.72, 0.81)	1.46 (1.27, 1.65)	0.94 (0.92, 0.96)
**Severity of hepatic steatosis (%)**									
None	7,940 (66.5)	7,940 (91.7)	0 (0)	7,940 (92.3)	0 (0)	7,940 (98.6)	0 (0)	0 (0)	0 (0)
Mild	1,695 (13.5)	387 (5.4)	1,308 (35.0)	270 (2.8)	1,425 (41.0)	62 (0.8)	325 (66.5)	208 (33.2)	1,100 (35.4)
Moderate	1,931 (13.6)	204 (2.4)	1,727 (43.0)	328 (3.3)	1,603 (40.1)	30 (0.6)	174 (27.4)	298 (43.0)	1,429 (43.0)
Severe	914 (6.4)	40 (0.5)	874 (22.0)	163 (1.6)	751 (18.8)	11 (0.1)	29 (6.1)	152 (23.8)	722 (21.6)
Diabetes (%)	1,852 (10.3)	767 (6.0)	1,085 (21.6)	957 (7.6)	895 (17.3)	767 (6.5)	0 (0)	190 (24.0)	895 (21.1)
Hypertension (%)	4,046 (27.9)	2,225 (20.9)	1,821 (46.4)	2,541 (24.5)	1,505 (36.7)	2,202 (22.3)	23 (2.7)	339 (57.2)	1,482 (44.3)
Metabolic syndrome (%)	4,113 (28.3)	1,848 (17.2)	2,265 (57.8)	2,186 (20.6)	1,906 (47.6)	1,834 (18.3)	0 (0)	352 (53.4)	1,906 (58.2)
History of myocardial infarction (%)^#^	395 (2.5)	203 (1.7)	192 (4.7)	225 (1.9)	170 (4.1)	201 (1.8)	2 (0.2)	24 (3.1)	168 (5.0)
History of congestive heart failure (%)^#^	328 (1.5)	176 (1.0)	152 (2.8)	191 (1.1)	137 (2.5)	173 (1.1)	3 (0.4)	18 (2.1)	134 (3.0)
History of stroke (%)^#^	208 (1.3)	113 (1.0)	95 (2.0)	121 (1.0)	87 (2.0)	112 (1.1)	1 (0.2)	9 (0.3)	86 (2.4)
Elevated liver enzymes (%)^#^	1,192 (7.6)	540 (4.9)	652 (14.5)	756 (6.7)	436 (9.8)	511 (4.9)	29 (4.8)	245 (31.8)	407 (11.0)
High alcohol consumption (%)^#^	1,026 (16.5)	682 (15.4)	344 (19.8)	1,026 (21.5)	0 (0)	682 (16.6)	0 (0)	344 (75.4)	0 (0)
Viral hepatitis (%)^#^	383 (2.6)	269 (2.5)	114 (2.9)	383 (3.6)	0 (0)	269 (2.7)	0 (0)	114 (17.3)	0 (0)
Iron overload (%) ^#^	407 (3.7)	306 (4.4)	101 (1.8)	407 (5.1)	0 (0)	306 (4.7)	0 (0)	101 (10.9)	0 (0)

* *Continuous values were presented as mean (95% confidence interval) and categorical variables were presented as counts (percentages), weighted by sample weights. Percentages may not total 100 due to rounding*.

# *The values were missing for some participants*.

*MAFLD, metabolic dysfunction-associated fatty liver disease; NAFLD, non-alcoholic fatty liver disease; Control, participants without MAFLD or NAFLD; N, participants only with NAFLD; M, participants only with MAFLD; M+N, participants with MAFLD and NAFLD; BMI, body mass index; SBP, systolic blood pressure; DBP, diastolic blood pressure; HbA1C, glycosylated hemoglobin; HOMA-IR, homeostasis model assessment-insulin resistance; TG, triglyceride; TC, total cholesterol; HDL, high-density lipoprotein cholesterol; AST, aspartate transaminase; ALT, alanine transaminase; GGT, gamma-glutamyl transferase; ALP, alkaline phosphatase; NFS, NAFLD fibrosis score; APRI, AST-to-platelet ratio index; FIB-4, Fibrosis-4 index*.

22.8% of participants were diagnosed with both NAFLD and MAFLD (M+N), and the weighted prevalence of only NAFLD (N) and only MAFLD (M) was 5.1 and 4.6% (Table 1). At baseline, group N were youngest (mean age: 34.2 years) and complicated with fewest metabolic disorders and histories of cardiovascular diseases. Among these four groups, group M had the highest proportion of men (66.7%), ever smokers (70.5%), the highest prevalence of high alcohol consumption (75.4%), viral hepatitis (17.3%), iron overload (10.9%), hypertension (57.2%), severe hepatic steatosis (23.8%) and the highest level of blood pressure (mean SBP: 129 mmHg; mean DBP:79.2 mmHg), liver enzymes (mean AST: 33.3 U/L; mean ALT: 31.0 U/L; mean GGT: 69.1 U/L; mean ALP: 88.7 U/L), and fibrosis scores (mean NFS score: −1.45; mean APRI score: 0.47; mean FIB-4 score: 1.46). Group M+N had the highest prevalence of metabolic syndrome (58.2%), with highest level of blood lipid (mean TG: 1.89 mmol/L, mean TC: 5.42 mmol/L), blood glucose (mean HbA1c: 5.66%).

### Associations of MAFLD/NAFLD With Mortality

We used LASSO regularization to preselect 11 covariates (Supplementary Table 2), of which age, sex, and race-ethnicity, hepatic assessment (FIB-4 score, NFS score, ALP, and CRP), metabolic parameters (WC, SBP, HbA1c, fasting insulin, TG) were selected for further adjustment. In univariable models, MAFLD increased the risk for all-cause mortality by one-fold compared with non-MAFLD participants. In reference to non-MAFLD participants, MAFLD enhanced the risk for all-cause mortality significantly when age, sex, race-ethnicity, FIB-4, NFS score, ALP, and CRP was adjusted [HR 1.21 (1.09, 1.33)], but this increase was non-significant when waist circumference, HbA1c, SBP, TG, and fasting insulin were further adjusted [HR 1.03 (0.93, 1.15)]. In reference to non-NAFLD participants, NAFLD increased the risk for all-cause mortality by around 50%, and the significance was lost after age, sex and ethnicity related factors were corrected [HR 1.05 (0.87, 1.28)] (Table 2). Both MAFLD and NAFLD showed a relatively significant positive correlation with cardiovascular and neoplasm mortality, however, risks of these mortalities were equal between participants with and without MAFLD or NAFLD after age and sex were adjusted. The relative risk of diabetes-related mortality was markedly elevated in participants with either MAFLD or NAFLD even after all factors were adjusted.

**Table 2:** Cox regression model for overall and disease-specific mortality of participants.

	**Deaths**	**Unadjusted HR**	**Model 1**	**Model 2**	**Model 3**
**Overall mortality**					
MAFLD	1,561	2.07 (1.86, 2.29)^*^	1.27 (1.16, 1.41)^*^	1.21 (1.09, 1.33)^*^	1.03 (0.93, 1.15)
NAFLD	1,326	1.47 (1.20, 1.79)^*^	1.05 (0.87, 1.28)	0.99 (0.81, 1.20)	0.81 (0.66, 1.00)
**Cardiovascular mortality**					
MAFLD	409	2.01 (1.66, 2.44)^*^	1.17 (0.96, 1.42)	1.10 (0.90, 1.34)	0.83 (0.68, 1.02)
NAFLD	352	1.53 (1.26, 1.86)^*^	1.07 (0.89, 1.30)	0.99 (0.81, 1.21)	0.80 (0.65, 0.98)^*^
**Neoplasm mortality**					
MAFLD	356	1.78 (1.45, 2.17)^*^	1.16 (0.94, 1.42)	1.12 (0.91, 1.39)	1.12 (0.88, 1.41)
NAFLD	307	1.31 (1.06, 1.61)^*^	1.01 (0.82, 1.25)	0.98 (0.79, 1.22)	0.96 (0.76, 1.21)
**Diabetes-related mortality**					
MAFLD	99	6.86 (3.94, 11.95)^*^	4.57 (2.63, 7.97)^*^	4.40 (2.49, 7.76)^*^	1.84 (0.97, 3.50)
NAFLD	78	3.26 (1.90, 5.59)^*^	2.54 (1.49, 4.34)^*^	2.72 (1.59, 4.63)^*^	1.38 (0.81, 2.37)
**Overall mortality**					
Control	2,139	Ref	Ref	Ref	Ref
N	73	0.48 (0.34, 0.68)^*^	0.92 (0.65, 1.31)	0.95 (0.65, 1.38)	1.09 (0.75, 1.58)
M	308	2.76 (2.28, 3.33)^*^	1.87 (1.57, 2.23)^*^	1.73 (1.44, 2.08)^*^	1.47 (1.22, 1.77)^*^
N+M	1,253	1.85 (1.65, 2.07)^*^	1.17 (1.05, 1.29)^*^	1.12 (1.00, 1.24)^*^	0.96 (0.86, 1.07)
**Cardiovascular mortality**					
Control	551	Ref	Ref	Ref	Ref
N	15	0.46 (0.20, 1.02)	1.01 (0.45, 2.30)	0.93 (0.36, 2.42)	1.24 (0.48, 3.25)
M	72	2.35 (1.60, 3.45)^*^	1.53 (1.03, 2.28)^*^	1.47 (0.98, 2.20)	1.05 (0.70, 1.58)
N+M	337	1.86 (1.51, 2.28)^*^	1.11 (0.91, 1.35)	1.03 (0.84, 1.27)	0.80 (0.64, 0.98)
**Neoplasm mortality**					
Control	530	Ref	Ref	Ref	Ref
N	21	0.46 (0.24, 0.89)^*^	0.81 (0.42, 1.56)	0.88 (0.46, 1.72)	0.89 (0.46, 1.72)
M	71	2.16 (1.50, 3.10)^*^	1.54 (1.08, 2.20)^*^	1.59 (1.12, 2.26)^*^	1.58 (1.09, 2.28)^*^
N+M	285	1.63 (1.31, 2.02)^*^	1.08 (0.87, 1.35)	1.04 (0.83, 1.31)	1.04 (0.81, 1.34)
**Diabetes-related mortality**					
Control	66	Ref	Ref	Ref	Ref
N	0	NA	NA	NA	NA
M	21	9.13 (4.15, 20.05)^*^	6.66 (3.03, 14.62)^*^	5.53 (2.61, 11.71)^*^	2.09 (0.71, 6.14)
N+M	78	5.86 (3.25, 10.58)^*^	4.01 (2.23, 7.22)^*^	4.02 (2.33, 6.94)^*^	1.78 (0.95, 3.35)

* *p <0.05*.

*Values were presented as hazard ratio (95% confidence interval). Model 1: adjusted by age, sex and race-ethnicity (N = 12,480). Model 2: Model 1+ adjusted by FIB-4 score, NFS score, CRP, ALP (N = 12,281). Model 3: Model 2 + adjusted by waist circumference, HbA1c, SBP, TG, fasting insulin (N = 12,281). HR, hazard ratio; MAFLD, metabolic dysfunction-associated fatty liver disease, compared with non-MAFLD participants; NAFLD, non-alcoholic fatty liver disease, compared with non-NAFLD participants; Control, participants without MAFLD or NAFLD; N, participants only with NAFLD; M, participants only with MAFLD; M+N, participants with MAFLD and NAFLD; NA, not applicable*.

**Figure 2:**
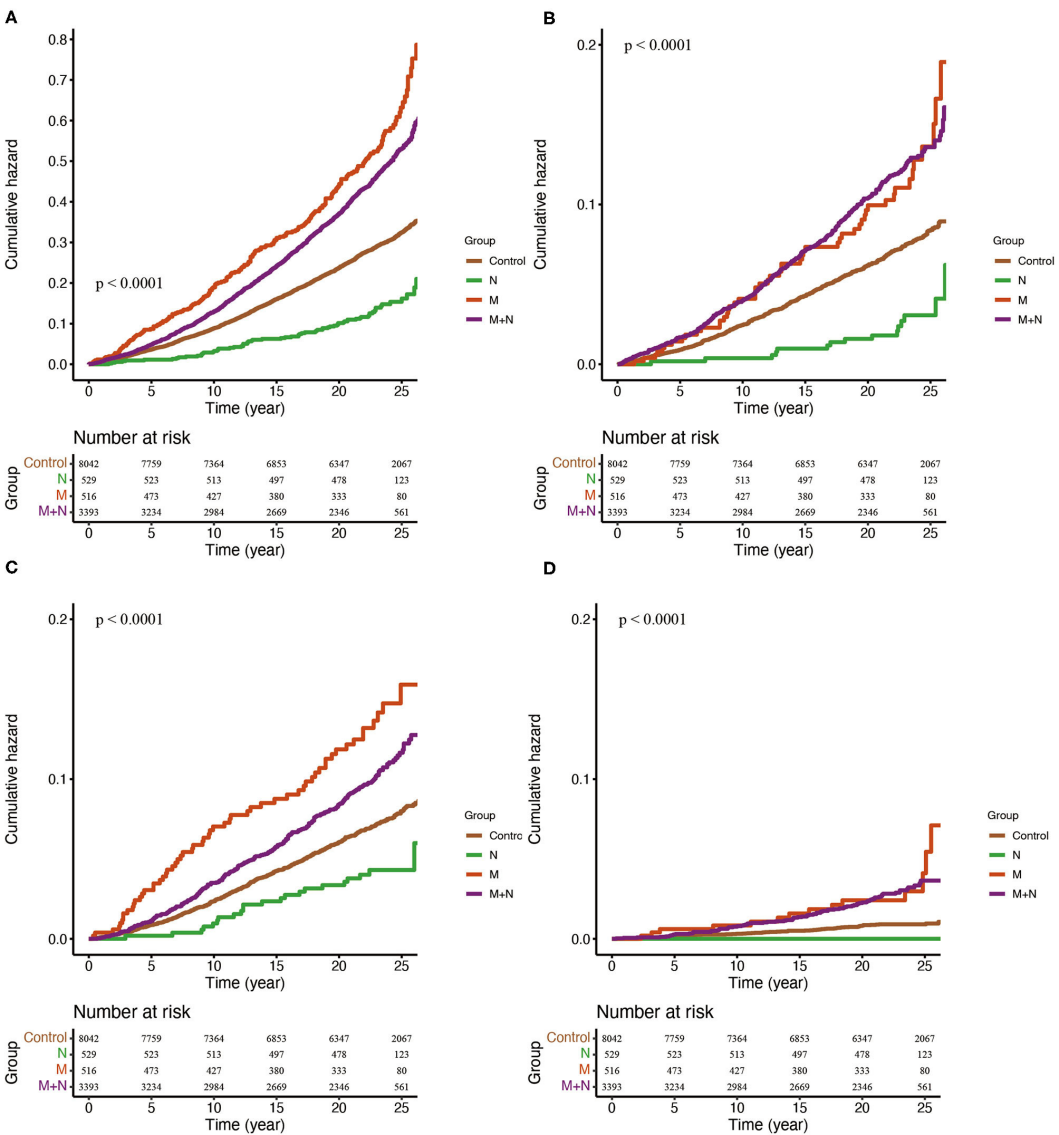
Kaplan-Meier estimates of overall **(A)**, cardiovascular **(B)**, neoplasm **(C)** and diabetes-related **(D)** mortality. Control, participants without MAFLD or NAFLD. N, participants only with NAFLD; M, participants only with MAFLD; M+N, participants with MAFLD and NAFLD.

We further divided the participants into four groups. In reference to the group without MAFLD and NAFLD (control group), group N reduced all-cause mortality by around 50%, and the association was non-significant after age, sex and race-ethnicity were adjusted; group M independently increased the risk of all-cause mortality by 47%; group M+N was significantly associated with elevated all-cause mortality unless waist circumference, HbA1c, SBP, TG and fasting insulin were adjusted [HR 0.96 (0.86, 1.07)] (Figure 2A, Table 2). For cardiovascular mortality, group M and group M+N both showed an increased risk than control in-crude, but this risk was unaltered in group M after FIB-4, NFS score, CRP and ALP score were adjusted and in group N+M after sex and age were adjusted (Figure 2B, Table 2). Group M independently increased the risk of neoplasm mortality after all confounders were adjusted. The risk of neoplasm mortality was reduced in group N and enhanced in group M+N in reference to control group in-crude (Figure 2C, Table 2). Group M and group M+N relatively enhanced risk of diabetes-related mortality unless corrected by metabolic factors, compared with the control group (Figure 2D, Table 2). The risk of group N in diabetes-related risk was unavailable without enough events.

### Subgroup Analysis of MAFLD

**Figure 3:**
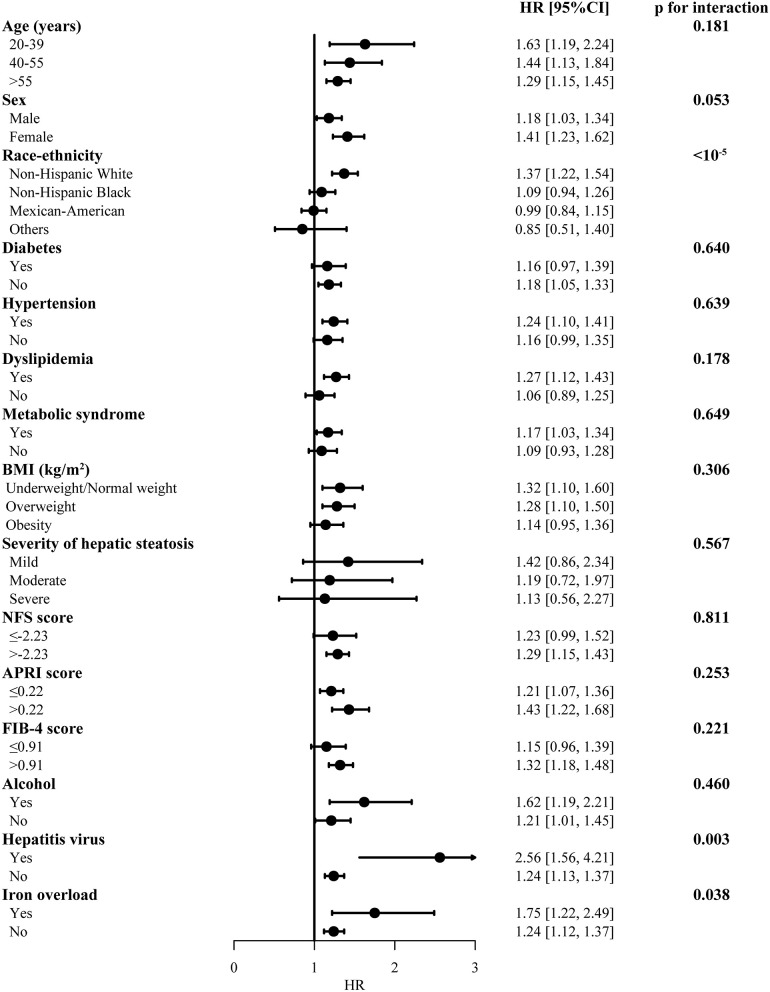
Subgroup analysis for the overall mortality in participants with MAFLD. The model was adjusted by adjusted by age, sex and race-ethnicity. MAFLD, metabolic dysfunction-associated fatty liver disease, compared with non-MAFLD participants; BMI, body mass index; HR, hazard ratio; CI, confidence internal. Significance was determined as *p* < 0.0033 (Bonferroni correction applied).

The risk of the MAFLD for overall mortality was similar in subgroups with different age, BMI, severity of hepatic steatosis, diabetes, hypertension, dyslipidemia, metabolic syndrome, FIB-4, and other etiologies (Figure 3). Significant heterogeneity was only found in different ethnicities and presence of viral hepatitis (Bonferroni corrected). MAFLD increased risk for all-cause mortality in non-Hispanic white race [HR 1.37 (1.22, 1.54)], with viral hepatitis [HR 2.56 (1.56, 4.21)] or without viral hepatitis [HR 1.24 (1.13, 1.37)]. There was no difference in subgroups in cardiovascular and neoplasm mortality risk in MAFLD (Supplementary Figures 1, 2).

## Discussion

Compared with patients with NAFLD, patients with MAFLD had increased risk for all-cause mortality in a greater magnitude in spite of similar cardiovascular, neoplasm and diabetes-related mortality risk. The nomenclature changes excluded participants who were negatively associated with mortality and captured participants who had higher all-cause mortality risk. The risk of mortality was similar among MAFLD subgroup except for non-Hispanic white race and viral hepatitis comorbidity.

Our study identified that patients diagnosed with new definition would have greater all-cause mortality risk in a medium follow-up time of 22.8 years. The risk for cardiovascular and neoplasm mortality was similar between MAFLD and NAFLD. Similarly, a previous study suggested that the fatal and non-fatal cardiovascular outcomes were similar between NAFLD and MAFLD after a 7-year follow-up (17). This indicated that the term MAFLD emphasized total mortality risk but did not affect the major outcomes of fatty liver. Adjusting confounders for mortality step by step, we found the association between NAFLD and all-cause mortality was non-significant after age and sex were adjusted and the risk of MAFLD on all-cause mortality was largely attributable to the dysregulated metabolic profile. The impact of metabolic disorder on mortality was more prominent in MAFLD compared with NAFLD. The risk of fatty liver on cardiovascular and neoplasm mortality was mainly owning to age, sex and race and our study showed cause-specific mortality was similar between NAFLD and MAFLD.

Some researchers were concerned that this new definition may lose some participants, especially those with severe steatosis (24). However, our study suggested in the patients excluded after the name switch, only 6% had severe steatosis. The excluded patients were mainly participants with NAFLD without apparent metabolic disorder, who had a “cardio-protective” metabolic profile as well as significantly lower liver enzymes and hepatic fibrosis scores. More importantly, they showed reduced risk with mortality possibly owing to their young age and low levels of metabolic disorder. Therefore, the participants excluded might not be the priority for clinical intervention and drug development.

Other researchers found that the change in terminology included more patients with metabolic disorders (14), these patients were included in group M in our study. This group was independently associated with all-cause mortality, especially with neoplasm mortality. They were identified with the worst metabolic profile and advanced hepatic inflammation and fibrosis, indicating possible worse liver outcomes (25, 26). These patients ignored by previous criteria of NAFLD were mainly patients with alcohol abuse, hepatic viral infection and iron overload, which tended to speed up the progression of extracellular and hepatocellular cancer (27). The risk for total mortality and cardiovascular mortality was similar between group M and group M+N, indicating the drugs developed for MAFLD may also be applicable for group M. Also, MAFLD with or without other etiology showed no heterogeneity in subgroup analysis regarding all-cause mortality risk. In this scenario, patients in group M may also benefit from drugs developed for MAFLD.

Similar HRs were observed in subgroups of age, sex, smoking status, metabolic dysfunction, hepatic steatosis, FIB-4, and different etiologies. We only detected significant heterogeneity of race-ethnicity and presence of hepatic viral infection in all-cause mortality, after Bonferroni correction (*p*_interaction_ < 0.05/13) was applied. The hazard ratio of MAFLD was highest in non-Hispanic white, followed by non-Hispanic black, Mexican-American and other races. As non-Hispanic whites accounted for over three fourth in the United States, they may be the group most affected by this disease. The design for clinical trials could possibly consider stratify patient recruitment according to ethnicities. We also observed significantly greater risks for overall mortality among patients with MAFLD secondary to hepatic viral infection, whereas failing tested the heterogeneity in cause-specific mortality. As the seventh leading cause of death globally and an increasing epidemic trend (28), hepatic virus infection may primarily accelerate the course of liver-related especially with comorbidity of fatty liver disease. Our results suggested drug development for patients with MAFLD should take racial difference and viral hepatitis infection into consideration in the future.

Our study used a large population-based prospective cohort with long follow-up to analyze the association between MAFLD/NAFLD and mortality. However, there are several limitations to our current study. The liver outcomes, especially fine categorization of liver cancer and advanced cirrhosis, were still needed for a comprehensive vision on the natural history of fatty liver disease. We were unable to perform the analysis due to data acquisition limitations. However, the incidence rate of cardiovascular mortality was around 5 times higher than liver-specific mortality in NAFLD (1). Extrahepatic neoplasm may be a primary source for extra mortality in NAFLD (5). In the NHANES III cohort, NAFLD showed similar liver-related mortality with non-NAFLD controls and liver-related mortality only account for <2% of total mortality (19, 20). Liver-related mortality become more relevant when the stages of steatosis progressed, however, this required precise categorization of fatty liver stages which we were unable to perform. The liver-related outcomes may change when other etiology, e.g., alcoholic liver disease (AFLD) and viral hepatitis, was included in MAFLD. Nevertheless, one study suggested in a fatty liver cohort with mixed background of NAFLD and AFLD, mortality from cardiovascular disease and total neoplasm still surpassed liver cirrhosis (29). In addition, the new definition emphasized the presence of metabolic derangements which mainly leads to elevated cardiovascular risk. By this means, we used total mortality, cardiovascular mortality and cancer mortality as our outcomes should still provide robust information to reveal the impact of nomenclature change. Secondly, hepatic steatosis in adults was detected by imaging techniques instead of liver histology, possibly weakening the reliability of the diagnosis of NAFLD. But one qualified meta-analysis showed high sensitivity and specificity in the detection of moderate-severe hepatic steatosis by ultrasound (30). With the improvement of ultrasound, imaging techniques still had limited sensitivity to detect mild steatosis (31). The study only used ultrasound results 30 years ago and the sensitivity of ultrasound detection was greatly improved in recent years (32). Thirdly, we did not excluded the drug-induced hepatotoxicity in the NAFLD definition since we were unable to establish causal relationship between drug use history and fatty liver in an epidemiological survey. One study reported very small portion of participants taken drugs related to hepatotoxicity, and there was no significant difference in mortality after excluding them (33). Finally, some non-statistically significant findings may be related to the limited sample size especially in subgroup analysis, indicative of lower power of the study. More similar studies should be designed and integrated to reduce type 2 error.

In conclusion, using baseline and follow-up data from the cohort of NHANES III, we found MAFLD had an enhanced risk for mortality and similar risk for cause-specific mortality with NAFLD. The definition MAFLD emphasized the role of metabolic disorder on the outcomes of fatty liver since the risk of MAFLD for mortality was largely attributable to its metabolic disorder. The switch from NAFLD to MAFLD captured participants with higher mortality risk regardless of losing some patients with reduced mortality risk. Ethnic differences and the presence of virus hepatitis should be taken into consideration when trials investigating outcomes for MAFLD were implemented.

## Data Availability Statement

Publicly available datasets were analyzed in this study. This data can be found here: https://www.cdc.gov/nchs/nhanes/nh3data.htm.

## Ethics Statement

The studies involving human participants were reviewed and approved by NCHS Ethics Review Board. The patients/participants provided their written informed consent to participate in this study.

## Author Contributions

QH, XZo, and LJ designed the research. QH and XZo collected, analyzed the data, and drafted the manuscript. XW and XZh revised the manuscript. All authors contributed to the article and approved the submitted version.

## Conflict of Interest

The authors declare that the research was conducted in the absence of any commercial or financial relationships that could be construed as a potential conflict of interest.
