# Supplementary material for: NAFLD or MAFLD: Which Has Closer Association With All-Cause and Cause-Specific Mortality?—Results From NHANES III
Source: Front Med (Lausanne). 2021 Jul 1;8:693507. doi: 10.3389/fmed.2021.693507 (PMC8280321; doi:10.3389/fmed.2021.693507)
Supplement: Supplementary file 1 [file Data_Sheet_1.docx]

Supplementary Material

**Supplementary Table 1 Formulas in the Study**

|  | Formulas | References |
| --- | --- | --- |
| **HOMA-IR** | Ins (mU/L) × FPG (mmol/L)/22.5 | ^1^ |
| **NFS** | −1.675 + 0.037 × age (years) + 0.094 × BMI (kg/m^2^) + 1.13 × IFG/diabetes (yes = 1, no = 0) + 0.99 × AST/ALT– 0.013 × platelet (×10^9^/L) – 0.66 × albumin (g/dL) | ^2^ |
| **APRI** | [AST/AST (ULN)]/ platelet (×10^9^/L) | ^3^ |
| **FIB-4** | (Age (years) × AST (U/L))/ ((PLT [10^9^/L]) × (ALT (U/L))^1/2^) | ^4^ |

HOMA-IR, homeostasis model assessment-insulin resistance. NFS, NAFLD fibrosis score. APRI, AST-to-platelet ratio index. FIB-4, Fibrosis-4 index. ULN, upper limit normal.

**Supplementary Table 2 Coefficients of selected variables in LASSO Cox regression model for all-cause mortality of participants**

| Variables | Coefficients |
| --- | --- |
| Age | 1.0934 |
| SBP | 0.1695 |
| CRP | 0.1599 |
| HbA1c | 0.1340 |
| Male | 0.1261 |
| FIB-4 | 0.0553 |
| TG | 0.0112 |
| ALP | 0.0019 |
| Waist circumference | 0.0015 |
| Fasting insulin | 0.0011 |
| NFS score | 0.0004 |

Partial likelihood deviance in the LASSO Cox regression model to analyze overall mortality among participants with complete covariates (N=12279). The variables were chosen by ten-fold cross-validation with minimum mean error.


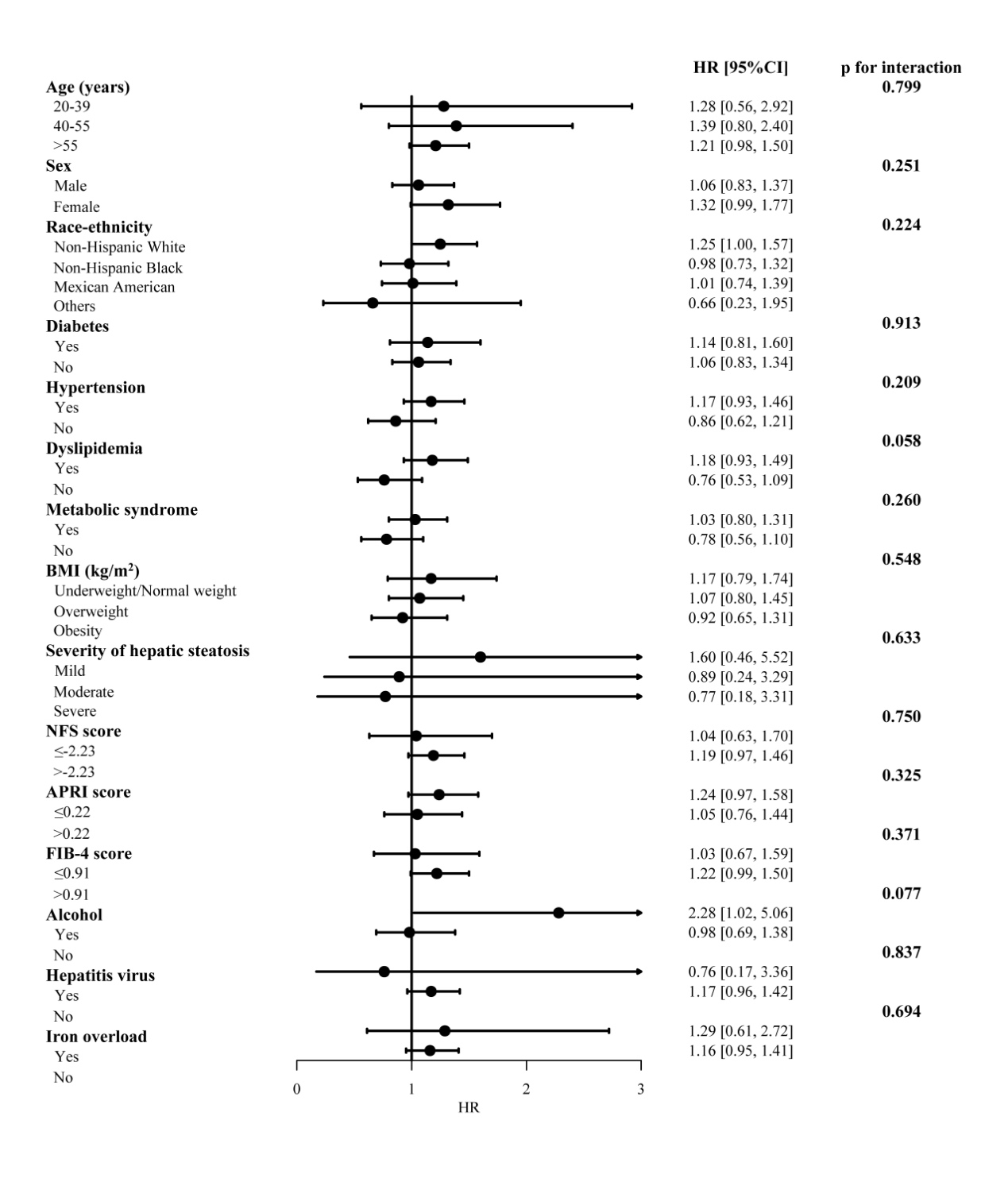


**Supplementary Figure 1 Subgroup analysis for the cardiovascular mortality in participants with MAFLD.**

The model was adjusted by adjusted by age, sex and race-ethnicity. MAFLD, metabolic dysfunction-associated fatty liver disease, compared with non-MAFLD participants. BMI, body mass index. HR, hazard ratio. CI, confidence internal. Significance was determined as p<0.005 (Bonferroni correction applied).

**
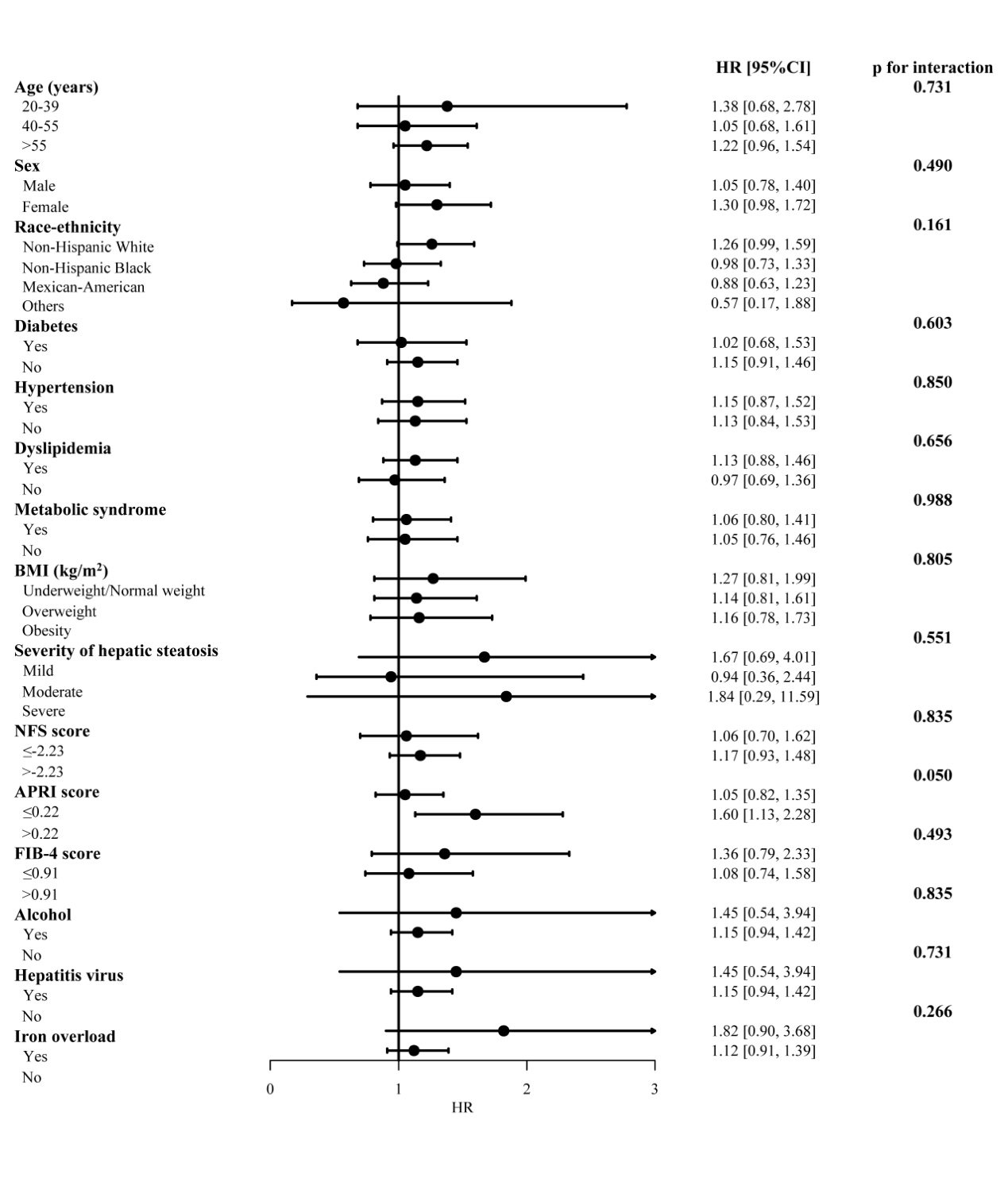
Supplementary Figure 2 Subgroup analysis for the neoplasm mortality in participants with MAFLD.** The model was adjusted by adjusted by age, sex and race-ethnicity. MAFLD, metabolic dysfunction-associated fatty liver disease, compared with non-MAFLD participants. BMI, body mass index. HR, hazard ratio. CI, confidence internal. Significance was determined as p<0.005 (Bonferroni correction applied).

**References**

1. Matthews DR, Hosker JP, Rudenski AS, Naylor BA, Treacher DF, Turner RC. Homeostasis model assessment: insulin resistance and β-cell function from fasting plasma glucose and insulin concentrations in man. *Diabetologia*. 1985;28:412–419.

2. Angulo P, Hui JM, Marchesini G, et al. The NAFLD fibrosis score: a noninvasive system that identifies liver fibrosis in patients with NAFLD. *Hepatology*. 2007;45:846–854.

3. Wai C-T, Greenson JK, Fontana RJ, et al. A simple noninvasive index can predict both significant fibrosis and cirrhosis in patients with chronic hepatitis C. *Hepatology*. 2003;38:518–526.

4. Vallet‐Pichard A, Mallet V, Nalpas B, et al. FIB‐4: an inexpensive and accurate marker of fibrosis in HCV infection. comparison with liver biopsy and fibrotest. *Hepatology*. 2007;46:32–36.
